# Health-Related Data Sources Accessible to Health Researchers From the US Government: Mapping Review

**DOI:** 10.2196/43802

**Published:** 2023-04-27

**Authors:** Ann Annis, Crista Reaves, Jessica Sender, Sherry Bumpus

**Affiliations:** 1 College of Nursing Michigan State University East Lansing, MI United States; 2 Institute for Health Policy Michigan State University East Lansing, MI United States; 3 University Libraries Michigan State University East Lansing, MI United States; 4 School of Nursing Eastern Michigan University Ypsilanti, MI United States

**Keywords:** data sets as topic, federal government, data collection, survey, questionnaire, health surveys, big data, government, data set, public domain, data source, systematic review, mapping review, review method, open data, health research

## Abstract

**Background:**

Big data from large, government-sponsored surveys and data sets offers researchers opportunities to conduct population-based studies of important health issues in the United States, as well as develop preliminary data to support proposed future work. Yet, navigating these national data sources is challenging. Despite the widespread availability of national data, there is little guidance for researchers on how to access and evaluate the use of these resources.

**Objective:**

Our aim was to identify and summarize a comprehensive list of federally sponsored, health- and health care–related data sources that are accessible in the public domain in order to facilitate their use by researchers.

**Methods:**

We conducted a systematic mapping review of government sources of health-related data on US populations and with active or recent (previous 10 years) data collection. The key measures were government sponsor, overview and purpose of data, population of interest, sampling design, sample size, data collection methodology, type and description of data, and cost to obtain data. Convergent synthesis was used to aggregate findings.

**Results:**

Among 106 unique data sources, 57 met the inclusion criteria. Data sources were classified as survey or assessment data (n=30, 53%), trends data (n=27, 47%), summative processed data (n=27, 47%), primary registry data (n=17, 30%), and evaluative data (n=11, 19%). Most (n=39, 68%) served more than 1 purpose. The population of interest included individuals/patients (n=40, 70%), providers (n=15, 26%), and health care sites and systems (n=14, 25%). The sources collected data on demographic (n=44, 77%) and clinical information (n=35, 61%), health behaviors (n=24, 42%), provider or practice characteristics (n=22, 39%), health care costs (n=17, 30%), and laboratory tests (n=8, 14%). Most (n=43, 75%) offered free data sets.

**Conclusions:**

A broad scope of national health data is accessible to researchers. These data provide insights into important health issues and the nation’s health care system while eliminating the burden of primary data collection. Data standardization and uniformity were uncommon across government entities, highlighting a need to improve data consistency. Secondary analyses of national data are a feasible, cost-efficient means to address national health concerns.

## Introduction

In today's digital-centric health care industry, data are ubiquitous from electronic health records and billing records to data warehouses and guideline clearing houses. Data influence our clinical decisions, practices, and policies. Unfortunately, primary data collection is both time intensive and costly. Given limited resources, conducting a large study may not be feasible for many researchers, and obtaining funding can be a formidable barrier. Therefore, researchers often seek secondary sources of data to support their research needs. National data sources in the United States include big data from surveys and data sets sponsored and managed by federal agencies. These national data sources may provide opportunities for researchers to build collaborative teams, gather preliminary data, access populations that may otherwise be inaccessible, and test their hypotheses in support of future work. These data sources can be used to observe important trends and disparities in health care experiences across populations [1,2]. Further, national data sources can provide insights into the efficiencies and effectiveness of the US health care system and provider workforce. Yet, navigating these sources can be challenging in terms of identifying the right data sources, understanding how the data were collected and what kind of information is available, and knowing how to access the data.

Despite the widespread availability of national data, there is little guidance for researchers on how to access and evaluate the use of these resources. Few publications have attempted to provide an overview of national data sources accessible to researchers. In 1 such review, Blewett et al [3] examined 6 major household surveys conducted by federal agencies. Their work presented an informative summary of national surveys; yet, the study was limited to 6, purposefully selected sources. They did not include other population-based, national data sources nor did they review data sources focused on health care providers and systems. Similarly, Cohen et al [4] described 5 national data sources that addressed health care delivery systems. Their critique was limited to data sources specifically used to evaluate delivery systems, and thus, was also not comprehensive.

The lack of cataloged, comprehensive information pertaining to publicly available, health-related data sets highlights a need for increased awareness and understanding of these resources, particularly for researchers planning to use these national data. We sought to address this gap by providing a broad overview of national data sources in the United States. Thus, our aim was to identify and summarize a comprehensive list of federally sponsored, health- and health care–related data sources that are accessible in the public domain in order to facilitate their use by researchers. By reviewing and cataloging national data sources, this review offers useful guidance to researchers for identifying relevant data sets and where to find them. This review provides a roadmap to national data sources, allowing researchers to include and better leverage these resources in their research projects.

## Methods

### Study Design

We conducted a systematic mapping review of federally sponsored sources of health care data in the United States. A mapping review is ideal when knowledge is broad enough that “categorizing, classifying, and characterizing patterns, trends or themes in evidence production” [5,6] is needed. We selected a mapping review methodology because systematic mapping, as opposed to a systematic review, does not require specific, a priori research questions [7]. Rather, systematic mapping is intended to describe and catalog available information on a topic area, permitting a broad assessment of the data. Evidence collected in a systematic mapping review is collated and organized in a database, providing detailed metadata (ie, a set of data that defines and describes data from specific sources) for each item under investigation [7].

This review was restricted to national data sources because they are representative of the US population’s health experiences and address a wide variety of topics, thus likely to appeal to a diverse cadre of health researchers. National data sources tend to involve large segments of the US population and yield estimates that are more robust and generalizable than smaller data sources. Additionally, these sources often benefit from multiyear funding, comprehensive and standardized protocols, rigorous methodologies, statistical support, and data user guides. Lastly, it was necessary to limit our search to a discrete population of data sources that were feasible to evaluate within the scope of this review and would likely be reproducible. All data collected for this review was publicly available via government websites; thus, institutional review board approval was not required.

### Data Sources and Search Criteria

A research librarian (JS) conducted an internet search to find relevant sources of national health care data. The search was constructed to identify government websites for publicly available surveys and data sets. Guided by a prior review of data access policies among federal and state data sources [8], we began with a similar initial search of the major federal agencies and subagencies involved in health research, including the Department of Health and Human Services, the Centers for Disease Control and Prevention (CDC), the National Institutes of Health, the Health Resources and Services Administration, the Food and Drug Administration, the Centers for Medicare & Medicaid Services (CMS), the Substance Abuse and Mental Health Services Administration, and the Agency for Healthcare Research and Quality (AHRQ). We also included the Bureau of Labor Statistics and the Census Bureau as potential sources of health-related data.

A multistep process was used to gain familiarity with the data sources and refine our search strategy. Our original search strategy was developed and conducted in 2019 and repeated in May of 2021. An exhaustive review of the designated agencies was undertaken to ensure all eligible data sets, and surveys associated with these government agencies were identified. First, Boolean search logic (survey OR surveys OR dataset OR data) was used on each website to discover data sets. Second, websites that had specific sections labeled as “data” or “research” were hand searched to identify other surveys and data collections affiliated with related agencies or organizations. We defined a data source as a digital location where data sets with a shared, common purpose and originating from the same entity are stored. A data set is a collection of data.

We included data sources that (1) are federally sponsored by a US government agency; (2) include data on US populations of interest, including individuals, patients, health care providers, health care sites and systems; (3) contain health- or health care-related data; and (4) have active and ongoing data collection, or if inactive, then the most recent year data collection was 2010 or later. Data sources were excluded if they did not meet the above criteria or if they (1) were limited to data on biological or genetic samples, radiologic images, laboratory tests or testing practices, (2) did not produce publicly available data sets, (3) referred only to a “parent” or family entity and not an individual data source, (4) only included aggregated statistics or reports, or (5) were duplicate records.

### Data Collection

For each data source, we identified the federal government agency responsible for producing the data and determined whether it belonged to a family. A data source “family” is a set of multiple data sources with distinct but related purposes managed by the same government agency (eg, Healthcare Cost and Utilization Project [HCUP]). Each member of a family was screened as an individual data source.

An iterative process was used to abstract and categorize data. We determined a core list of variables to collect from each data source, which was informed by prior reviews [3,4] as well as by our own knowledge and experience working with national data. A spreadsheet was developed to document our review process, details of the review, and to systematically collect the core variables. Four separate data abstractions were conducted, whereby team members (AA, CR, and SB) were each randomly assigned 5 data sources from which to abstract and record information. At the onset, it was evident that due to the nature of embedded website design, question-specific URLs in addition to the data source’s home page URL would need to be collected. This facilitated our ability to conduct redundant reviews and in-depth analyses as warranted.

The team discussed findings from data abstractions during weekly meetings, and the core variables were expanded and modified, as needed, based on consensus. When revisions were made, we rereviewed the data sources to ensure that the data previously collected accurately reflected the new, revised variable definitions and criteria. To ensure interrater reliability, team members reexamined at least 10% of all completed reviews by having a second, different abstractor perform a review, and then findings or discrepancies were further discussed during the weekly meetings. In this process, 1 team member would conduct the initial data extraction and review, and another member would ensure the completeness and accuracy of the data. For variables requiring categorization (eg, purpose of the data and population of interest), both reviewers would independently code the variables. A simple percent agreement was calculated as the number of rated items with an agreement between the 2 reviewers divided by the total number of rated items for a particular data source. Any variables with disagreement were reassessed and discussed until 100% agreement was attained for all variables.

### Key Measures and Coding Scheme

Our data abstraction process collected information on 5 key domains from each data source: overview and purpose, population of interest and sampling design, methodology of data collection, type and description of data, and cost of data. To analyze these domains, we used a modified, data-based, convergent synthesis method [9]. This method allowed us to simultaneously summarize the data both quantitatively and qualitatively prior to synthesis. For each domain, we developed a coding schema, which was derived from our qualitative analysis, or predefined categories commonly used by the data sources, or a combination of the two (blended). A comprehensive list of definitions and coding schemas is provided in Table 1.

**Table 1:** Content domains, definitions, coding schemes, and categories of the 8 key measures used to classify features of the data sources included in the review.

Domains, their definitions, and categories		Category descriptions and examples
**Overview and purpose^a^: statements from the data sources’ websites that explain the underlying rationale for why these data are/were collected, what purposes the data serve, and how and by whom the data are used. (Coding scheme: qualitative process)**		
	Summative processed data	Data that are processed or summarized in a manner to allow for easier interpretation and use of the information, such as aggregated data files, statistical reports, and summaries. Common verbs used to describe these data sources: produce, create, inform, and yield
	Primary registries	Data collected from individuals who share a common condition or similar characteristics. Common verbs used to describe these data sources: collect, capture, compile, cover, and obtain.
	Survey/assessment data	Data collected directly from the population of interest to assess the current state of events, such as patient surveys. Common verbs used to describe these data sources: assess, describe, explore, survey, gather, and collect
	Evaluative data	Data to generate new knowledge or draw conclusions about particular issues. Common verbs used to describe these data sources: identify, understand, recognize, examine, evaluate, investigate, determine, and learn
	Trend data	Data that monitors or tracks specific events or conditions over time using repeated measures (not necessarily from the same sample population). Common verbs used to describe these data sources: track, measure, monitor, trend, and chronicle
**Population of interest^a^: the population being studied or assessed. Data reflect attributes or information originating from the population of interest. (Coding scheme: predefined categories)**		
	Persons	Individuals and patients
	Health care providers	Doctors, nurses, etc
	Health care systems	Hospitals, ambulatory care facilities, provider offices, etc
**Methodology of data collection: information from the data sources’ websites that describes characteristics of the data collection process. (Coding scheme: predefined categories)**		
	Frequency of data collection and data set release	Annual and biannual
	Timing of data collection	The most recent year for which data are available
	Mode of data collection	Web-based survey, in-person paper survey, telephone or in-person interview, and electronic data submission
	Who reported data	The individuals or entities who provided or reported the data
**Type of data^a^: a categorization of the types of data available in a data set. (Coding scheme: blended)**		
	Patient demographics	Age, sex, race, ethnicity, marital status, occupation, housing, education, health insurance
	Patient health-related behaviors and social history	Smoking, substance use, diet and exercise, health screening, and immunizations
	Patient clinical data	Medical history, electronic health record clinical data, medical procedures, health conditions, or diseases
	Laboratory data	Laboratory data
	Provider/practice characteristics	Descriptors of providers, provider workforce, or practice sites, such as years in practice, specialty, counts of providers, tasks performed, ownership and size of practice, geographic location, and use of staff
	Health care costs and expenditures	Costs of medical care, billing, or claims data
**Cost of data: information on the cost for researchers to access the data. (Coding scheme: blended)**		
	Free	Any of the available data sets are free
	Cost	All available data sets have a fee to access

^a^Domains where coding categories are not mutually exclusive.

### Overview and Purpose of the Data

We examined the overview and purpose descriptions available on the websites. A qualitative process was used to analyze these narrative descriptions and identify relevant themes. Similar verb usage was evident across data sources that shared similar purposes and became the foundation for coding these segments. We grouped comparable verbs together and applied an iterative process to compare and contrast data sources to categorize the objectives of each data source. Five general themes were identified (Table 1). Each data source was categorized into 1 or more themes accordingly.

### Population of Interest, Sampling Design, and Sample Size

For each data source, we identified the population of interest and sampling design. We distinguished the population of interest from those reporting the data (ie, respondents), which were not always identical. The sample size of the most recent data set available was determined, and data sources were grouped by quartile of their sample sizes.

### Methodology of Data Collection

We examined information pertaining to how the data was obtained from the population of interest, including the mode of data collection (eg, survey, interviews, and electronic submission), instruments used, frequency of data collection and data set release, and the most recent year of data available.

### Type and Description of Data

Classification of the type of data was guided by predefined, commonly reported categories [3] and informed by a review of variable definitions and coding information obtained from the data sources. Binary flags were created for each category (Table 1) to indicate the presence or absence of each type of data in a data set.

### Cost of Data

For each data source, it was determined whether free data sets were available and whether any fees were charged to researchers to obtain specific data sets.

## Results

### Overview of the Data Sources

**Figure 1:**
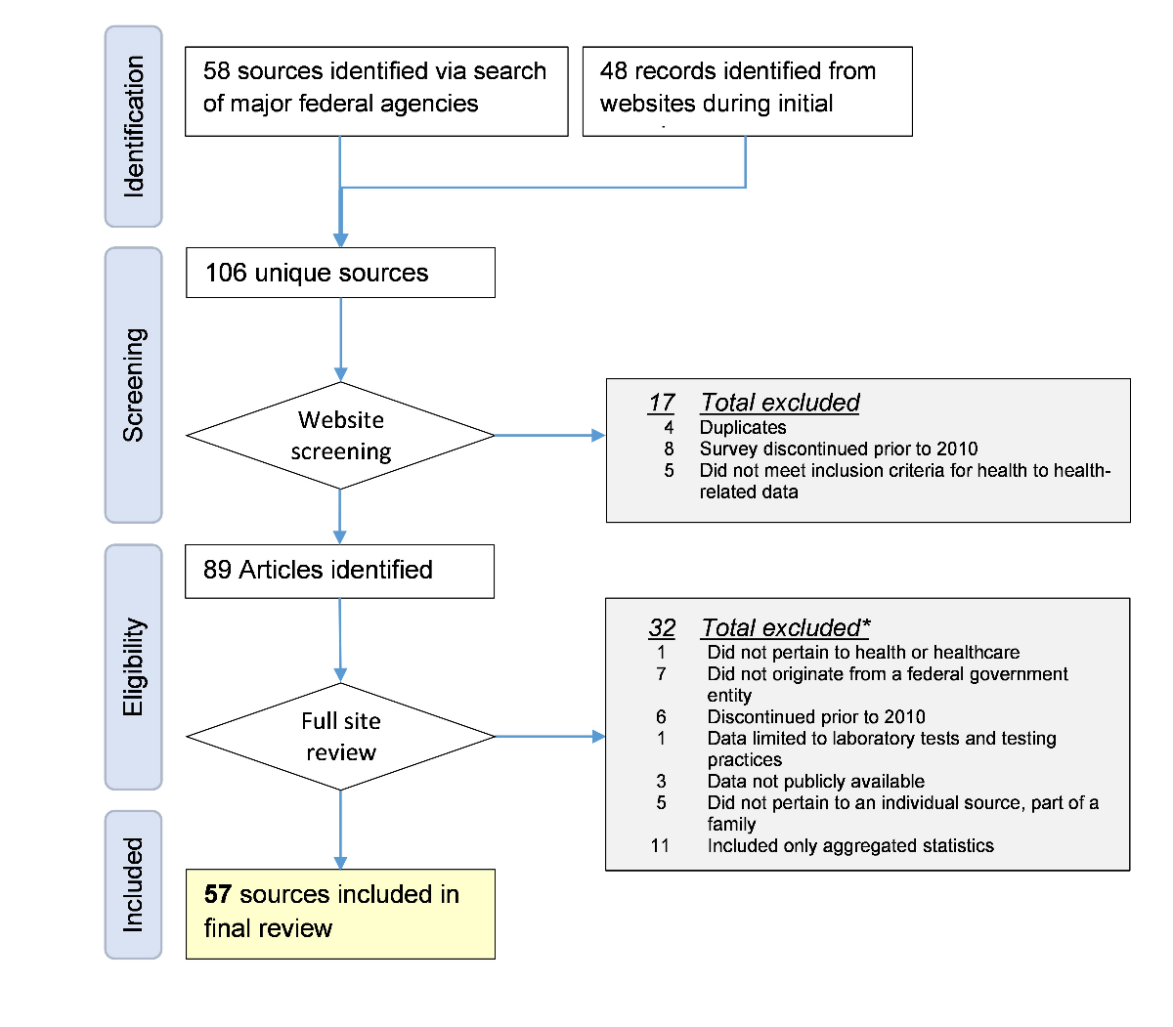
Flow diagram for the mapping review. *Articles can meet multiple exclusion criteria; thus, categories will not sum to total.

We identified a total of 106 eligible data sources (Figure 1). Our initial screening resulted in the removal of several data sources (n=17), and after further screening and exclusions (n=32), the final sample consisted of 57 unique data sources. Three major federal government agencies oversee the entities responsible for these data sources (Multimedia Appendix 1). The Department of Health and Human Services represented the largest share, covering 7 different entities and 52 data sources. We identified 8 families of related data sources. Multimedia Appendix 2 provides a summary and description of the 57 data sources, including key variables of interest.

### Key Measures

#### Overview and Purpose of the Data

Most data sources functioned as survey or assessment data (30/57, 53%) or provided summative processed data (27/57, 47%) and trends data (27/57, 47%), while fewer were primary registries (17/57, 30%) and evaluative data (11/57, 19%; Table 2). Most (39/57, 68%) were categorized as having more than 1 general purpose. For example, the Behavioral Risk Factor Surveillance System is the largest continuously conducted health survey system in the United States, monitoring national trends, and providing summarized data on health-related risk behaviors of US population, such as smoking and alcohol use [10].

**Table 2:** Frequencies for select measures collected for the review.

Domain and category (total unique data sources)		Number of data sources (n=57), n (%)
**Purpose^a^**		
	Code 1: summative processed data	27 (47)
	Code 2: primary registries	17 (30)
	Code 3: survey and assessment data	30 (53)
	Code 4: evaluative data	11 (19)
	Code 5: trends data	27 (47)
**Population of interest^a^**		
	Persons, individuals, and patients	40 (70)
	Health care providers	15 (26)
	Health care sites and systems	14 (25)
**Sample size**		
	0-11,999	13 (23)
	12,000-49,999	14 (25)
	50,000-999,999	14 (25)
	>1,000,000	14 (25)
**Types of data^a^**		
	Patient demographics	44 (77)
	Patient clinical data	35 (61)
	Patient health-related behaviors and other social histories	24 (42)
	Provider or practice characteristics	22 (39)
	Health care costs or expenditure data	17 (30)
	Laboratory data	8 (14)
**Cost**		
	Any of the available data sets are free	43 (75)
	All available data sets have fee to access	14 (25)

^a^Categories are not mutually exclusive, that is, a data source can be classified in more than 1 category.

#### Population of Interest, Sampling Design, and Sample Size

The sample population for the majority of data sources (40/57, 70%) was individuals or patients (Table 2). Fewer data sources focused on information related to health care providers (15/57, 26%) or health care systems (14/57, 25%). Many data sources used complex, multistaged, stratified sampling designs to obtain samples representative of larger US population groups, and thus permit the calculation of national estimates. For example, the National Ambulatory Medical Care Survey multistaged sampling includes geographically defined areas, hospitals within these areas, clinics within these hospitals, and patient visits to these clinics [11].

Several data sources also used oversampling of specific populations to improve the likelihood of producing valid estimates for groups that tend to be poorly represented in national data. The Health Information National Trends Survey purposefully oversamples individuals from minority populations to increase the number of participants from these groups, and thus improve precision in the estimates [12]. The sampling designs of many data sources were intended to be representative of the US population. Only a few sources were focused on a specific or narrowly defined population group. For example, the National Practitioner Data Bank collects all reports (not a sample) of medical malpractice from reporting entities. Sample sizes of data sets ranged from 637 (Compendium of US Health Systems [13]) to over 100,000,000 (National Death Index [14]), with approximately half having sample sizes larger than 50,000. Data sets from CMS vary in size, depending on the specific requests from researchers.

#### Methodology of Data Collection

The majority (36/57, 63%) of data sources produced annual data sets, while 25% (14/57) offered data sets every 2 years, and fewer used other data set release periods. Most (49/57, 86%) data sources were active with ongoing data collection. All of the data sources had delays between data collection and availability of data sets for the public (ie, no real-time data available). There was variation in data collection methodologies and how methods were described. For example, some well-established sources with data collection spanning decades, such as the National Health and Nutrition Examination Survey, have extensive documentation on methodology, data collection procedures, and survey instruments available to researchers on their websites [15].

Data sources that collected data via the administration of surveys were primarily cross-sectional, with a few notable exceptions. The National Longitudinal Surveys of the Youth used a longitudinal methodology, continuing to collect data every 2 years from an original sample recruited in the late 1970s and 1980s [16]. Similarly, the Health and Retirement Study (HRS) is a longitudinal panel study that collects repeated measures every 2 years from regularly recruited samples [17]. Data sources that maintained registries of health care events, such as the Nationwide Emergency Department Sample [18] and the Nationwide Inpatient Sample [19], could also potentially yield longitudinal information on individuals with multiple events, depending on the ability to link records for unique persons both within and across data set releases.

#### Type and Description of Data

A large majority of data sources collected data on individual demographics (44/57, 77%) and clinical information (35/57, 61%; Table 2). Fewer sources contained data on health-related behavior and social history (24/57, 42%), provider or practice characteristics (22/57, 39%), health care costs and expenditures (17/57, 30%), or laboratory tests (8/57, 14%). Twelve (21%) of the 57 data sources focused entirely on health care providers or practice. This included the Surveys on Patient Safety Culture, which are a family of surveys administered to staff in clinical sites [20].

#### Cost of Data

Most data sources (43/57, 75%) offered at least some deidentified data sets at no cost, while fewer data sources (14/57, 25%) charged a fee for available data sets (Table 2). Free data sets typically consisted of data aggregated at a group level and were available as deidentified public use files that were readily accessible to the general public for download. Data sets for purchase provided more granular data, requiring additional privacy protections and authorization, such as a formal application, protocol review, approval process, and a data use agreement. Data sets for purchase ranged from US $30 to thousands of dollars and could vary based on the type of file, the size of the cohort needed, the number of linked data sets required, the number of years or time period needed, the method of accessing the data (eg, data file sent electronically to researcher vs researcher has time-limited access to data via a secure web portal, requiring annual renewal fees), and the individual requesting the data (eg, students are often charged a reduced fee). Thus, due to the variability in data fees, we do not report specific costs and instead recommend that researchers consult directly with the data owners who are qualified to determine estimated costs for particular projects.

#### Unique Data Source: Medicare

The CMS produces a vast number of data sources pertaining to the Medicare population and Medicare providers [21]. As such, it was beyond the scope of this project to comprehensively summarize each CMS data source individually. Thus, we chose to present these sources as one resource. According to the CMS website [22], data are available for Medicare program use and payments, provider characteristics and initiatives, and beneficiary characteristics. Because the Medicaid program is managed by individual states, it did not meet inclusion criteria.

Several Medicare data sources are accessible directly from the CMS website. In addition, CMS contracts with the Research Data Assistance Center [23] at the University of Minnesota to provide data access and technical assistance to researchers with specific CMS data requests. Data sets are offered in 3 formats, based on the level of detail and customization needed: free public use file (aggregated data), purchasable limited data sets (individual level, deidentified data), and more costly research identifiable files (individual-level, customizable data). Because of the sheer number of data sources produced by CMS, they span a number of different purposes, populations, and methods.

## Discussion

### Principal Results

We summarized a comprehensive list of federally sponsored health-related data sources that are accessible to researchers (Multimedia Appendix 2). From this mapping review, 3 major findings are apparent that have implications for research. First, there is a vast amount of health-related big data available, accelerated by nationwide efforts to promote transparency and replicability in research studies. The wealth of accessible health data opens the door to a multitude of research opportunities. Big data can be used to identify important patterns and trends in health care, potentially improving care and decreasing costs [24]. These data can support a wide range of investigations into health issues, such as nutritional health, behavioral health, mental health, communicable infections, chronic conditions, occupational health, provider practice, quality of care, health services usage, and costs. Moreover, national data sources offer several advantages over primary data collection. Government-produced data sets are typically standardized and cleaned prior to release, thereby enhancing data quality. Large, nationally representative samples can enable the examination of rare events, which may not otherwise be feasible. Secondary data sources eliminate the financial burden of primary data collection—a clear advantage to researchers with limited funding.

Second, it is easier to select a data source first and then ask a question, rather than asking a question first and finding a data source to answer it. A common limitation of secondary data is that the data were originally collected for other purposes, and thus may not adequately or completely address a particular research question. In essence, the numerous data sources we identified would support a broad range of relevant research questions. However, attempting to fit a predetermined research agenda or line of inquiry to pre-existing data sources may be challenging. For example, research that aims to track patients at intervals throughout the course of a hospital admission would not be successful using a hospital admissions data set that does not contain repeated measures or does not allow linkages of unique patients longitudinally. Such a dilemma would require modification of the research goals or an alternative data source. Understanding whether specific variables of interest are available, and knowing how and why the data were collected are critical and can impact analyses and interpretation of findings [2].

Third, we note a lack of structure and standardization of data across national data sources, limiting integration. Although several of the long-running data sources had well-developed methodologies and standardization for their routinely released data sets, this was not always true. For example, the Surveys on Patient Safety Culture is a family of data sets managed by AHRQ that collect key information about patient safety reported by clinical sites. Although the central website [20] provides an overview and general information for each survey, there are limited resources that describe a detailed methodology and analytic considerations for researchers. A list of variables and definitions for the surveys is not readily available. Conversely, the HCUP, also managed by AHRQ, is a family of databases that collect information on health care events such as hospitalizations and surgeries occurring in medical facilities. The HCUP website [25] offers numerous resources to guide researchers in using the data sets, including file specifications documents, summary statistics for select variables, descriptions of data elements, detailed methodology documentation, and statistical software programming code.

In addition, there is an overall lack of structure and consistency in the manner in which data sets and related materials are made available, and there is a lack of cataloging of available data sources and corresponding data sets. For example, the CDC data sets are structured in an accessible and exemplary format where the researcher can navigate the overview, purpose, methodology, and survey results directly from the webpage. In contrast, the Surveys on Patient Safety Culture data sets require the researcher to find the needed information through various links embedded within the webpage. Not all data sources provided clear purpose or overview statements, descriptions of sampling and data collection methodologies, or guidance for researchers related to data acquisition and analyses. There was no unified organization as to how to access a copy of the survey (if applicable), data abstraction form, or data dictionary, or where to look to determine if there was a cost involved. This need for consistency in big data has been described by Wilkinson et al [26] in the FAIR data principles: findable (F), accessible (A), interoperable (I), and reusable (R).

### Implications for Research

It is likely that many of the data sources we reviewed are not fully leveraged by researchers. A lack of knowledge of how to find, access, and use these data sources may be a barrier—an issue this review intended to address. Multimedia Appendix 2 offers a starting point for researchers seeking a data source. We were encouraged to find that several government agencies offered not only extensive web-based materials, such as data dictionaries and data user guides but also provided workshops and training. For example, the HCUP databases have a comprehensive collection of free, technical assistance resources, training tools, and web-based tutorials [27]. Increasing the availability of such resources for data users is a key first step in promoting the use of these data sources.

National sources of health data are often used to report general descriptive information on populations and health. However, well-designed studies of secondary data with rigorous methodologies can provide valuable clinical information as well, such as through comparative effectiveness analyses of treatment options, which can complement clinical trials and inform clinical decision-making [24]. Furthermore, advanced big data analytic approaches, such as machine learning, now permit robust analyses beyond traditional observational methods [28]. Predictive modeling and proactive identification of at-risk populations are popular applications of such approaches.

Yet, in order to optimize the full value of big data, there is a need to adopt standards to ensure data quality, improve standardization across data sources to facilitate linkages, develop consistent data-sharing policies, and allow more timely access to data [29]. A shared set of guidelines for presenting data source information would further facilitate usage by researchers. In this space, we recognize current efforts nationally to promote the standardization of national data sources. For example, the Patient-Centered Outcomes Research Institute has developed multiple standards that support improved standardization of national data, providing guidance for data registries and networks [30]. Recommendations from the Patient-Centered Outcomes Research Institute standards include clearly defining the purpose and data elements of the data source, and using appropriate data quality checks and validation methods [30].

Optimal standardization of the metadata across data sources is not an easy task and, depending on the unique features of the data sources, may not be feasible under all circumstances. However, as evident in our review, some data sources have already developed processes by which to link data files with those from other government entities. For example, upon approval, HRS (supported by National Institutes of Health) provides data files linked to Medicare data (managed by CMS). Similarly, linked files are available for the National Health Interview Survey (supported by CDC) and the Medical Expenditure Panel Survey (maintained by AHRQ). These linked data files across government agencies will, by necessity, require some standardization of data elements to create such combined data sets. The continued development of these data linkages will likely improve data standardization. Furthermore, the ability to integrate data sources can help enhance and validate research findings. However, definitions of common data elements may vary across data sets. Thus, the use of common variables in linked data files should be carefully considered, as they may impact the way in which sources can be used together [4]. Improving and increasing the accessibility of linked data will further expand the use of national data.

Other key considerations for researchers include recognition of the limitations of these data sources. Long lag times between data collection and release—often in years—are inherent in secondary data sources. This is problematic for research involving temporally sensitive events, such as the COVID-19 pandemic. To our knowledge, none of the data sources we reviewed permitted real-time data access—the logistics of which would be challenging. However, compressing the time window for data set release would improve researchers’ access to more timely data. In addition, the inability to follow distinct individuals longitudinally, due to the lack of unique identifiers that can link subjects across multiple time periods or data sets is common among national data sources [2]. This prevents the examination of repeated measures and long-term outcomes. Although a few data sources we reviewed did have the capability to support longitudinal analyses (eg, HRS, Medicare data), most did not.

Our work provides an overview of national data sources that should be useful to those wanting to leverage secondary data sources for their research. The use of systematic mapping methodology for this review offers advantages. A mapping review allowed for a comprehensive assessment of national data sources that was not limited to any singular research topic area or focus. Mapping reviews are also intentionally structured to produce data sufficient to facilitate their use in future studies [7]. However, as a mapping review, it functions as a snapshot in time, identifying key federal agencies and the types of data sets currently available. Our overarching purpose was to provide meaningful information that could guide health researchers in their selection and potential use of these valuable data resources in their research endeavors. Although we did not originally plan to routinely update our list of data sources, the processes we undertook could be replicated in a future review. This would require updating recent changes to existing data sources and reviewing and including new sources as they emerge. In fact, this review provides the framework and tools needed to examine new federally sponsored data sets as they become available in the future.

### Limitations

Given the vast number of government agencies that collect health-related data, it is possible that we overlooked sources that should have been included in this review. We intentionally did not include nonfederal government data sources, including private and nonprofit organizations and state-based data sources, as these would have been too numerous to examine and beyond the scope of this review. It should also be noted that the sheer volume of information and number of web links associated with many government entities presents challenges when sorting through search results to identify relevant information. Although the search was undertaken systematically, we may have missed important information due to inconsistent and diverse information-sharing conventions of the data sources.

### Conclusions

In conclusion, this review offers insight into the availability of large, federally funded sources of health data. These data sources provide important measures of our nation’s health and health care system. Big data from these resources can support a wide range of research efforts, including preliminary or pilot work, evidence for clinical decision-making, trends analyses, and outcome studies. However, further advancements are needed in the field of big data to catalog available data and to unify data set presentation conventions and facilitate retrieval of important aspects, such as variable definitions. For now, these resources still provide academicians, clinicians, and researchers options at a lower cost and with more efficiency than primary prospective studies.

## Appendix

Multimedia Appendix 1 Associated government agencies and departments for the 57 data sources in the mapping review.

Multimedia Appendix 2 Selected characteristics of the 57 data sources included in the mapping review.

## Abbreviations

AHRQ: Agency for Healthcare Research and Quality
CDC: Centers for Disease Control and Prevention
CMS: Centers for Medicare & Medicaid Services
HCUP: Healthcare Cost and Utilization Project
HRS: Health and Retirement Study
