# Supplementary material for: Health-Related Data Sources Accessible to Health Researchers From the US Government: Mapping Review
Source: J Med Internet Res. 2023 Apr 27;25:e43802. doi: 10.2196/43802 (PMC10176148; doi:10.2196/43802)
Supplement: Multimedia Appendix 1 [file jmir_v25i1e43802_app1.pdf]

Department of Health and Human Services

AHRQ

1. Compendium of U.S. Health Systems
2. Consumer Assessment of Health Plan Survey (CAHPS)- Clinician and Group Survey Database
3. CAHPS- Health Plan Survey Database
4. Kids Inpatient Database
5. Medical Expenditure Panel Survey
6. National Inpatient Sample
7. Nationwide Ambulatory Surgery Sample
8. Nationwide Emergency Department Sample
9. Nationwide Inpatient Sample
10. Nationwide Readmissions Database
11. Survey on Patient Safety Culture (SOPS)- Ambulatory Surgery Center Database
12. SOPS Community Pharmacy Database
13. SOPS Hospital Database
14. SOPS Medical Office Database
15. SOPS Nursing Home Database
16. State Ambulatory Surgery and Services Databases
17. State Emergency Department Databases
18. State Inpatient Databases

CMS

1. Home Health Outcome and Assessment
2. Medicare Current Beneficiary Survey
3. Medicare data files

FDA

1. Population Assessment of Tobacco and Health

HRSA

1. Area Health Resources File
2. National Practitioner Data Bank
3. National Sample Survey of Nurse Practitioners
4. National Sample Survey of Registered Nurses
5. Uniform Data System

CDC

1. Behavioral Risk Factor Surveillance System
2. Household Pulse Survey
3. National Ambulatory Medical Care Survey
4. National Death Index
5. National Electronic Health Records Survey
6. National Health and Nutrition Examination Survey
7. National Health Interview Survey
8. National Hospital Ambulatory Medical Care Survey
9. National Hospital Care Survey
10. National Immunization Survey- Child
11. National Immunization Survey- Teen
12. National Study of Long-Term Care Providers
13. National Survey of Children with Special Health Care Needs
14. National Survey of Children’s Health
15. National Survey of Family Growth
16. Youth Risk Behavior Surveillance System

NIH

1. Health and Retirement Study
2. Health Information National Trends Survey
3. Monitoring the Future
4. Surveillance, Epidemiology, and End Results

SAMHSA

1. Drug Abuse Warning Network
2. National Mental Health Services Survey
3. National Survey of Substance Abuse Treatment Services
4. National Survey on Drug Use and Health
5. Treatment Episode Data Set

Department of Commerce

Census Bureau

1. American Community Survey

Department of Labor

Bureau of Labor Statistics

1. American Time Use Survey
2. National Longitudinal Survey of the Youth (1997)
3. National Longitudinal Survey of the Youth (1979)
4. National Longitudinal Survey of the Youth (1979) Child and Young Adult
