# Supplementary material for: Health-Related Data Sources Accessible to Health Researchers From the US Government: Mapping Review
Source: J Med Internet Res. 2023 Apr 27;25:e43802. doi: 10.2196/43802 (PMC10176148; doi:10.2196/43802)
Supplement: Multimedia Appendix 2 [file jmir_v25i1e43802_app2.pdf]

A. Annis, C. Reaves, J. Sender, S. Bumpus. (2023) U.S. government, health-related data sources accessible to health researchers: A mapping review.
Appendix 2. Characteristics of the national data sources included in the review (n=57)

										Purpose					Type of data available						Population focus		
Government Entity	Division	Name	Dataset family	Website (all sites active as of 04-12-2021)	Dataset year(s)	Sample	Sample size	Freq.	Cost	Summ.	Registry	Survey	Eval.	Trends	Demo.	Social	Clinical	Lab	Provider	Financial	Person	Provider	System
Department of Commerce	Census Bureau	American Community Survey (ACS)		https://www.census.gov/programs-surveys/acs/	2018	Subset of U.S. Census. Residents of U.S., Puerto Rico.	3,500,000	annual	free	✓		✓			✓	✓	✓				✓		
Department of Health and Human Services	AHRQ	Compendium of U.S. Health Systems		https://www.ahrq.gov/chsp/data-resources/compendium.html	2021	U.S. health systems	637	every 2 years	free		✓								✓	✓			✓
	AHRQ	Medical Expenditure Panel Survey (MEPS)		https://meps.ahrq.gov/mepsweb/	2018	Individuals from households	10,610	annual	free	✓			✓		✓	✓				✓	✓		
	AHRQ	Consumer Assessment of Healthcare Providers and Systems (CAHPS)-Clinician and Group Survey Database	Consumer Assessment of Healthcare Providers and Systems (CAHPS)	https://cahpsdatabase.ahrq.gov/CGSurveyGuidance.aspx	2019	Individuals enrolled in a managed care health plan who had a visit with a provider, practice, or medical group	330,390	every 6 months	free			✓			✓				✓			✓	
	AHRQ	Consumer Assessment of Healthcare Providers and Systems (CAHPS)-Health Plan Survey Database	Consumer Assessment of Healthcare Providers and Systems (CAHPS)	https://www.ahrq.gov/cahps/index.html	2019	Individuals enrolled in a managed care health plan	387,350	annual	free			✓			✓		✓		✓		✓	✓	✓
	AHRQ	Kids Inpatient Database (KID)	Healthcare Cost and Utilization Project (HCUP)	https://www.hcup-us.ahrq.gov/kidoverview.jsp	2016	Hospital inpatient stays for patients younger than 21 years	3,000,000	every 3 years	cost	✓	✓			✓	✓		✓		✓	✓	✓		
	AHRQ	National Inpatient Sample	Healthcare Cost and Utilization Project (HCUP)	https://www.hcup-us.ahrq.gov/nisoverview.jsp	2017	All-payer hospital inpatient stays	7,000,000	annual	cost	✓	✓			✓	✓		✓		✓	✓	✓		
	AHRQ	Nationwide Ambulatory Surgery Sample (NASS)	Healthcare Cost and Utilization Project (HCUP)	https://www.hcup-us.ahrq.gov/	2017	Ambulatory surgery encounters that do not result in an inpatient admission	7,600,000	annual	cost	✓	✓				✓		✓			✓	✓		
	AHRQ	Nationwide Emergency Department Sample (NEDS)	Healthcare Cost and Utilization Project (HCUP)	https://www.hcup-us.ahrq.gov/	2017	Emergency department visits that do not result in inpatient admission	33,500,000	annual	cost	✓	✓				✓		✓			✓	✓		
	AHRQ	Nationwide Inpatient Sample (NIS)	Healthcare Cost and Utilization Project (HCUP)	https://www.hcup-us.ahrq.gov/nisoverview.jsp	2018	Hospital inpatient stays	7,000,000	annual	cost	✓	✓				✓	✓	✓			✓	✓		
	AHRQ	Nationwide Readmissions Database (NRD)	Healthcare Cost and Utilization Project (HCUP)	https://www.hcup-us.ahrq.gov/nrdoverview.jsp	2018	Hospital inpatient discharges	18,000,000	annual	cost	✓	✓				✓		✓			✓	✓		✓
	AHRQ	State Ambulatory Surgery and Services Databases (SASD)	Healthcare Cost and Utilization Project (HCUP)	https://www.hcup-us.ahrq.gov/	2018	Ambulatory surgeries and other outpatient services from hospital-owned facilities	60,000,000	annual	cost	✓	✓				✓		✓			✓	✓		

										Purpose					Type of data available						Population focus		
Government Entity	Division	Name	Dataset family	Website (all sites active as of 04-12-2021)	Dataset year(s)	Sample	Sample size	Freq.	Cost	Summ.	Registry	Survey	Eval.	Trends	Demo.	Social	Clinical	Lab	Provider	Financial	Person	Provider	System
	AHRQ	State Emergency Department Databases (SEDD)	Healthcare Cost and Utilization Project (HCUP)	https://www.hcup-us.ahrq.gov/	2018	Emergency department visits that do not result in an inpatient admission	54,000,000	annual	cost	✓	✓			✓	✓		✓			✓	✓		
	AHRQ	State Inpatient Databases (SID)	Healthcare Cost and Utilization Project (HCUP)	https://www.hcup-us.ahrq.gov/	2019	Hospital inpatient discharges	17,686,332	annual	cost	✓	✓			✓	✓		✓			✓	✓		
	AHRQ	Surveys on Patient Safety Culture (SOPS)-Ambulatory Surgery Center Database	Surveys on Patient Safety Culture (SOPS)	https://www.ahrq.gov/sops/databases/asc/index.html	2020-2021	Ambulatory surgery centers	10,527	every 2 years	free			✓	✓	✓					✓			✓	✓
	AHRQ	Surveys on Patient Safety Culture (SOPS)-Community Pharmacy Database	Surveys on Patient Safety Culture (SOPS)	https://www.ahrq.gov/sops/surveys/pharmacy/index.html	2014-2018	Pharmacies	2,157	every 2 years	free			✓	✓	✓					✓			✓	✓
	AHRQ	Surveys on Patient Safety Culture (SOPS)-Hospital Database	Surveys on Patient Safety Culture (SOPS)	https://www.ahrq.gov/sops/databases/hospital/index.html	2018-2020	Hospitals	87,856	every 2 years	free			✓		✓					✓			✓	✓
	AHRQ	Surveys on Patient Safety Culture (SOPS)-Medical Office Database	Surveys on Patient Safety Culture (SOPS)	https://www.ahrq.gov/sops/databases/medical-office/index.html	2017-2019	Medical offices	18,396	every 2 years	free	✓		✓							✓			✓	✓
	AHRQ	Surveys on Patient Safety Culture (SOPS)-Nursing Home Database	Surveys on Patient Safety Culture (SOPS)	https://www.ahrq.gov/sops/databases/nursing-home/index.html	2016-2018	Nursing homes	10,499	every 2 years	free	✓		✓							✓			✓	✓
	CDC	Household Pulse Survey		https://www.census.gov/programs-surveys/household-pulse-survey.html	2021	Individuals from households	59,000	bi-weekly	free	✓		✓		✓	✓	✓	✓				✓		
	CDC	National Death Index (NDI)		https://www.cdc.gov/nchs/ndi/index.htm	2020	All death records	100,000,000	annual	cost		✓				✓		✓				✓		
	CDC	National Electronic Health Records Survey		https://www.cdc.gov/nchs/nehrs/about.htm	2017	Office-based physicians	301,603	annual	free	✓				✓					✓			✓	✓
	CDC	National Health Interview Survey (NHIS)		https://www.cdc.gov/nchs/nhis/about_nhis.htm	2019	Individuals from households	39,000	annual	cost			✓	✓	✓	✓	✓	✓				✓		
	CDC	National Health and Nutrition Examination Survey (NHANES)		https://www.cdc.gov/nchs/nhanes/index.htm	2017-2020	Individuals from households	27,066	every 2 years	free	✓		✓		✓	✓	✓	✓	✓			✓		
	CDC	National Study of Long-Term Care Providers		https://www.cdc.gov/nchs/nsltcp/index.htm	2018	Adult day services centers and residential care communities	7,414	every 2 years	free	✓				✓	✓		✓		✓			✓	
	CDC	National Survey of Children with Special		http://www.cdc.gov/nchs/slaits/cshcn.htm	2011	Children with special needs from households	40,242	every 4 years	free			✓	✓		✓		✓				✓		

										Purpose					Type of data available						Population focus		
Government Entity	Division	Name	Dataset family	Website (all sites active as of 04-12-2021)	Dataset year(s)	Sample	Sample size	Freq.	Cost	Summ.	Registry	Survey	Eval.	Trends	Demo.	Social	Clinical	Lab	Provider	Financial	Person	Provider	System
		Health Care Needs																					
	CDC	National Survey of Children's Health (NSCH)		http://www.cdc.gov/nchs/slaits/nsch.htm	2018	Households with at least one child 0-17 years	38,141	annual	free	✓					✓	✓	✓				✓		
	CDC	National Survey of Family Growth		https://www.cdc.gov/nchs/nsfg/nsfg_questionnaires.htm	2017-2019	Individuals 15-49 years	11,347	every 2 years	free			✓			✓		✓				✓		
	CDC	Behavioral Risk Factor Surveillance System (BRFSS)	Behavioral Risk Factor Surveillance System (BRFSS)	https://www.cdc.gov/brfss/	2019	Adults 18 years or older	400,000	annual	free	✓		✓		✓	✓	✓	✓				✓		
	CDC	Youth Risk Behavior Surveillance System (YRBSS)	Behavioral Risk Factor Surveillance System (BRFSS)	https://www.cdc.gov/healthyyouth/data/yrbs/index.htm	2017	9th through 12th grade students	4,400,000	annual	free	✓			✓	✓	✓	✓					✓		
	CDC	National Ambulatory Medical Care Survey (NAMCS)	National Health Care Surveys	https://www.cdc.gov/nchs/ahcd/index.htm	2017	Visits to office-based physicians and community health centers	9,953	annual	free			✓			✓		✓	✓			✓		
	CDC	National Hospital Ambulatory Medical Care Survey (NHAMCS)	National Health Care Surveys	https://www.cdc.gov/nchs/ahcd/index.htm	2018	Visits to emergency departments, outpatient departments, and ambulatory surgery locations	860,386	annual	free			✓			✓		✓		✓		✓		
	CDC	National Hospital Care Survey (NHCS)	National Health Care Surveys	https://www.cdc.gov/nchs/nhcs/index.htm	2016	Hospitals with six or more inpatient beds	44,500,000	annual	cost	✓				✓	✓		✓	✓			✓		
	CDC	National Immunization Survey-Child	National Immunization Survey	https://www.cdc.gov/vaccines/imz-managers/nis/about.html	2018	Parents and guardians of children in households	28,971	annual	free	✓	✓				✓	✓					✓		
	CDC	National Immunization Survey-Teen	National Immunization Survey	https://www.cdc.gov/vaccines/imz-managers/nis/about.html	2018	Parents and guardians of adolescents aged 13-17 years in households	39,000	annual	free	✓	✓				✓	✓	✓				✓		
	CMS	Home Health Outcome and Assessment (OASIS)		https://www.cms.gov/Medicare/Quality-Initiatives-Patient-Assessment-Instruments/HomeHealthQualityInits/OASIS-Data-Sets	2018	Medicare and Medicaid patients 18 years or older	.	annual	free			✓			✓		✓		✓		✓		✓
	CMS	Medicare Current Beneficiary Survey (MCBS)		https://www.cms.gov/Research-Statistics-Data-and-Systems/Research/MCBS	2018	Medicare beneficiaries	15,237	annual	free			✓	✓	✓	✓	✓	✓			✓	✓		
	CMS	Medicare and Medicaid data files		https://resdac.org/	2020	Medicare beneficiaries, health care providers, health care systems	.	annual	free	✓		✓		✓	✓	✓	✓	✓	✓	✓	✓	✓	✓
	FDA	Population Assessment of Tobacco and Health (PATH)		https://www.fda.gov/tobacco-products/tobacco-science-research/research	2018-2019	Individuals 9 years or older in households	45,971	every 2 years	free			✓	✓	✓	✓	✓	✓	✓			✓		
	HRSA	Area Health Resources File		https://data.hrsa.gov/topics/health-workforce/ahrf	2019-2020	Health care practitioners	980,000	annual	free	✓		✓							✓			✓	

										Purpose					Type of data available						Population focus		
Government Entity	Division	Name	Dataset family	Website (all sites active as of 04-12-2021)	Dataset year(s)	Sample	Sample size	Freq.	Cost	Summ.	Registry	Survey	Eval.	Trends	Demo.	Social	Clinical	Lab	Provider	Financial	Person	Provider	System
	HRSA	National Practitioner Data Bank		https://data.hrsa.gov/data/download https://www.npdb.hrsa.gov/topNavigation/aboutUs.jsp	2020	Health care practitioners	65,000	annual	cost		✓				✓		✓		✓	✓		✓	
	HRSA	National Sample Survey of Nurse Practitioners (NSSNP)		https://data.hrsa.gov/topics/health-workforce/nursing-workforce-survey-data	2012	Actively licensed Nurse Practitioners	13,000	once	free			✓							✓			✓	
	HRSA	National Sample Survey of Registered Nurses (NSSRN)		https://www.census.gov/nssrn	2018	Registered Nurses in the workforce	50,273	every 4 years	free				✓	✓					✓			✓	
	HRSA	Uniform Data System (UDS)		https://data.hrsa.gov/tools/data-reporting	2020	HRSA-funded health centers and primary care clinics	1,375	annual	free	✓		✓		✓	✓	✓	✓	✓	✓	✓			✓
	NIH	Health Information National Trends Survey (HINTS)		https://hints.cancer.gov/	2017	Adults 18 years or older	7,674	annual	free			✓	✓	✓	✓	✓					✓		
	NIH	Health and Retirement Study (HRS)		http://hrsonline.isr.umich.edu/	2018	Adults 51 years or older	26,000	every 2 years	free			✓		✓	✓	✓	✓	✓		✓	✓		
	NIH	Monitoring the Future		http://www.monitoringthefuture.org	2018	Students in 8th, 10th, 12th grade, college, and young adulthood	50,000	annual	free			✓	✓		✓	✓					✓		
	NIH	Surveillance, Epidemiology, and End Results (SEER)		https://seer.cancer.gov/	2017	Cancer cases reported from population-based cancer registries	696,950	annual	free		✓				✓		✓	✓			✓		
	SAMHSA	Drug Abuse Warning Network	Substance Abuse and Mental Health Data Archive	https://www.datafiles.samhsa.gov/data-sources	2011	Emergency department visits involving recent drug use	229,211	ended 2011	free					✓	✓	✓						✓	
	SAMHSA	National Mental Health Services Survey	Substance Abuse and Mental Health Data Archive	https://www.samhsa.gov/data/data-we-collect	2019	Mental health treatment facilities	11,866	annual	free		✓								✓	✓			✓
	SAMHSA	National Survey of Substance Abuse Treatment Services (N-SSATS)	Substance Abuse and Mental Health Data Archive	https://www.samhsa.gov/data/data-we-collect	2019	Facilities that provide substance abuse treatment	15,961	annual	cost		✓								✓				✓
	SAMHSA	National Survey on Drug Use and Health	Substance Abuse and Mental Health Data Archive	https://www.samhsa.gov/data/data-we-collect	2019	Individuals 12 years or older from households	67,507	annual	free			✓				✓					✓		
	SAMHSA	Treatment Episode Data Set (TEDS)	Substance Abuse and Mental Health Data Archive	https://www.samhsa.gov/data/data-we-collect	2018	Individuals 12 years or older who were admitted to facility for substance abuse treatment	1,864,367	annual	free					✓	✓	✓					✓		
Department of Labor	Bureau of Labor	American Time Use		https://www.bls.gov/tus/	2019	Individuals 15 years or	40,500	annual	free	✓					✓						✓		

										Purpose					Type of data available						Population focus		
**Government Entity**	**Division**	**Name**	**Dataset family**	**Website (all sites active as of 04-12-2021)**	**Dataset year(s)**	**Sample**	**Sample size**	**Freq.**	**Cost**	**Summ.**	**Registry**	**Survey**	**Eval.**	**Trends**	**Demo.**	**Social**	**Clinical**	**Lab**	**Provider**	**Financial**	**Person**	**Provider**	**System**
	Statistics	Survey				older																	
	Bureau of Labor Statistics	National Longitudinal Survey of the Youth (1979)	National Longitudinal Surveys	https://www.bls.gov/nls/nlsy79.htm	2016	Individuals born between 1957-1964 who participated in 1979 survey	12,686	every 2 years	free			✓		✓	✓	✓	✓				✓		
	Bureau of Labor Statistics	National Longitudinal Survey of the Youth (1997)	National Longitudinal Surveys	https://www.bls.gov/nls/nlsy97.htm	2018	Individuals born between 1980-1984 who participated in 1997 survey	8,984	every 2 years	free			✓		✓	✓	✓	✓				✓		
	Bureau of Labor Statistics	National Longitudinal Survey of the Youth 1979 Child and Young Adult	National Longitudinal Surveys	https://www.bls.gov/nls/nlsy79.htm	2016	Children born to mothers who participated in National Longitudinal Survey of the Youth 1979	11,543	every 2 years	free			✓		✓	✓	✓	✓				✓		

Agency abbreviations: AHRQ= Agency for Healthcare Research and Quality; CDC= Centers for Disease Control and Prevention; CMS= Centers for Medicare & Medicaid Services; FDA= Food and Drug Administration; HRSA= Health Resources & Services Administration; NIH= National Institutes of Health; SAMHSA= Substance Abuse and Mental Health Services Administration

Abbreviations: Summ= summative; Eval= evaluative; Demo= demographics;Freq= frequency of data file production
